# Understanding Factors Which Help and Hinder Engagement with Weight Management Interventions and Related Research

**DOI:** 10.3390/bs16071231

**Published:** 2026-07-20

**Authors:** Tanisha Douglas, Elizabeth Linforth, Michelle Lee, Jeffrey Stephens, Laura Wilkinson

**Affiliations:** 1 School of Psychology, Faculty of Medicine, Health and Life Science, Swansea University, Singleton Park, Swansea SA2 8PP, UKm.d.lee@swansea.ac.uk (M.L.); j.w.stephens@swansea.ac.uk (J.S.); l.l.wilkinson@swansea.ac.uk (L.W.); 2 Department of Psychology and Counselling, Birmingham City University, C358 Curzon Building, Birmingham B4 7BD, UK

**Keywords:** weight management, healthcare, engagement, attrition, recruitment

## Abstract

Those engaged in healthcare and research within the context of weight management report persistent difficulties with engagement. The aim of the current study was to identify factors which help or hinder engagement in weight loss interventions and associated research. Participants (*N* = 98) who were currently attempting or had recently attempted to lose weight by a diversity of means (including ‘going it alone’) were recruited through an online platform. Participants were asked to complete an online questionnaire with closed- and open-ended questions assessing the factors which helped and hindered their engagement. The results of the reflexive thematic analysis of open-ended responses generated three themes related to (1) components of the intervention (e.g., goal-setting tools and peer support), (2) commitment influences (e.g., time), and (3) motivational influences (e.g., emotional experiences). Our findings resonate with key components of behaviour change approaches and potentially support their use in the context of improving engagement with weight loss interventions and associated research. Future studies should take inventive approaches to understanding barriers to engagement amongst very disengaged individuals.

## 1. Introduction

Increasing overweight and obesity rates remain a global health concern, with the latest World Health Organization (WHO) findings relating that, as of 2022, one in eight people were living with obesity, as defined by body mass index (BMI). Compared to the prevalence of obesity in 1990, this has doubled for adults and quadrupled for adolescents (World Health Organization, 2025). A plethora of interventions (i.e., diet and exercise interventions and surgeries) have been recommended to address this, including strategies focused on engagement; however, evidence for achieving sustained weight loss is limited (Wolfenden et al., 2019). ‘Engagement’ refers to a range of concepts that are relevant at different stages of weight management intervention, including initiation, attendance, adherence, drop-out and attrition (Nobles et al., 2018). There is considerable variability in the reporting of engagement within the weight management intervention literature. With respect to disengagement, there is a substantial variation reported across interventions, ranging from 10 to 80% (Moroshko et al., 2011). Considering this, it has been suggested that when engagement is low, this will be reflected in the intervention outcomes. For example, in a cohort of participants with an average BMI of 34 (indicating obesity), it was found that those who consistently engaged (compared with ‘some’ and ‘minimal’) reported lower weight regain (Funk et al., 2010). Several studies have sought to understand factors that influence engagement with weight loss intervention. A rapid review of 11 studies (qualitative, quantitative, and mixed methods) identified several factors which increased digital intervention engagement; these were as follows: personalization, social support, feedback and encouragement, ease of set-up and use, inclusion of self-monitoring tools and prompts, and accessibility of information and knowledge (Sharpe et al., 2017). In comparison, studies addressing engagement with non-digital interventions have focused on demographics as predictors (Funderburk et al., 2016), individual psychological traits, such as self-efficacy (Linde et al., 2006), and practical barriers such as scheduling constraints (McVay et al., 2018).

When research accompanies a weight loss intervention, additional factors influence engagement. For example, in the context of bariatric–metabolic surgery, Gourash et al. (2016) reviewed 44 articles and found that research engagement decreased with more invasive procedures and increased with study compensation, and that participation in research follow-up may increase clinical follow-up, though no consistent demographic or psychosocial predictors were identified.

The aim of the current study was to draw on a diversity of approaches to weight loss intervention to further understand engagement and to consider the relatively under-researched area of engagement with research associated with weight loss interventions A qualitative survey-based approach was used, with open-ended questions employed in order to reduce barriers to participation (particularly scheduling, as a questionnaire can be completed at the participants’ convenience). Individuals who were currently attempting or had recently attempted to lose weight, with the inclusion of attempts via any means, were included in this study. Finally, building on Gourash et al. (2016), the survey questions about engagement with both the intervention itself and research associated with an intervention were included.

## 2. Materials and Methods

### 2.1. Participants

Participants were recruited online via social media groups that were tailored to weight loss, using the postings of a researcher (with an administrator’s permission). Participants were offered the opportunity to be entered into a prize draw with a chance of winning one of two £25 Amazon.com vouchers. Participants’ anonymity was preserved even if they provided their email address to enter the prize draw as we unlinked this information from their data. We advised participants via the information and consent form that to participate, they should be over 18 years old, and currently or recently engaged in a weight-loss attempt by any method. The criteria ensured that a broad and diverse range of perspectives were captured in the research. Individuals who had a previous or current eating disorder were not eligible to participate as this population would require a more tailored intervention, which does not align with the aims of this research.

Ethical approval was granted by the Swansea University School of Psychology Research Ethics Committee (Project ID: 1403). The study was conducted between March and September 2019.

An initial 146 responses were recorded; 98 of those responses were complete and included confirmation that the respondent was currently attempting or had recently attempted to lose weight. Incomplete responses were excluded; this was defined as participants who were under the age of 18 (*n* = 3), participants who did not consent (*n* = 1), participants who were not making a weight-loss attempt (*n* = 2) and participants who did not provide a response (*n* = 42). The sample size is consistent with the minimum suggested by Braun and Clarke (2013) of 80–100 respondents for a qualitative questionnaire study. The sample consisted of 85 females, 12 males and 1 who did not report their gender. Participants were aged between 18 and 68 years old; mean and standard deviation are reported in Table 1. The mean BMI of participants (*N* = 95, due to 3 missing values) was 29.75 ± 6.5.

### 2.2. Measures

**Survey Questions.** Drawing on current themes from across the extant literature, five questions (i.e., motivations for engagement (Sharpe et al., 2017) and type of weight loss intervention (Gourash et al., 2016)) were presented to the participants to explore their engagement with their chosen weight-loss approach and any factors or circumstances that they thought might have affected this. To facilitate understanding, we provided a brief explanation of what we meant by the term ‘engagement’, including brief descriptions of initiation, attendance and adherence. We also explained that research is often undertaken to evaluate a weight-loss intervention. Full question text is shown in Table 2.

Closed-ended questions were used to collect demographic information (self-reported gender, age, height and weight), the nature of the approach taken to weight-loss (i.e., commercial weight-loss plan, bariatric–metabolic surgery, going it alone or other approach), a quantitative measure of engagement with weight-loss attempt and previous experience/willingness to engage in weight-loss intervention research.

#### Procedure

Participants accessed the study by clicking an anonymous questionnaire link (Qualtrics, Provo, UT, USA). An information sheet was provided followed by an informed consent screen. Following confirmation of consent to participate, demographic questions were asked (age, gender, height, weight and heaviest weight outside of pregnancy). In addition, participants were asked to describe their approach to their current or recent attempt to lose weight. The engagement questions outlined above were then presented, followed by a debrief screen and an opportunity to enter the prize draw.

### 2.3. Data Analysis

Descriptive statistics were used to summarize responses to quantitative questions. This included calculating the participant’s BMI using the NHS online BMI calculator. Open-ended questions were analysed using thematic analysis (Braun & Clarke, 2006). This approach was chosen due its theoretical flexibility, which enables greater versatility for interpretation of patterns compared to other approaches to the analysis of qualitative data (Clarke & Braun, 2022). Two researchers undertook data analysis (TD and EL) independently and then compared results to determine agreement (investigator triangulation) This allowed the researcher to develop, challenge and refine the themes (Denzin, 1970; Thurmond, 2001).

Both in post-graduate roles, TD and EL had a theoretical knowledge of the approach and some practical experience; therefore, the analysis was supported by LW, who had supervisory responsibility. Discrepancies were discussed and resolved by the researchers and the third researcher (LW) was consulted if a resolution was not reached in the first instance.

In brief, six steps were followed by the two researchers: to (1) read and re-read the data for familiarity, (2) generate descriptive labels (codes) to reflect the data elements, (3) generate themes and sub-themes to encompass the codes, (4) refer back to the raw data to ensure the themes are reflective, (5) define and name the themes (at this point, the two analysts reviewed their analyses together to inform theme development) and, (6) produce the report while continuing to review the integrity of the themes (Braun & Clarke, 2006).

## 3. Results

Approach to weight loss. The majority of participants reported ‘going it alone’ for their weight loss attempt (42%), followed by a mainstream commercial weight management programme (36%). Non-commercially guided approaches were also reported (see Table 3).

The majority of participants described themselves as ‘engaged’ (34%) or ‘somewhat engaged’ (27%) (see Table 4) and just over half of the participants (53%) expressed a willingness to participate in research associated with a weight loss intervention.

### Thematic Analysis of Open-Ended Questions

**Figure 1:**
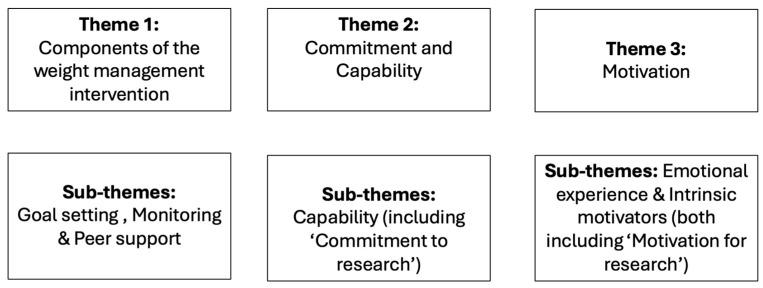
Structure of themes from the analysis.

Three themes were generated from the data which describe the participants’ (1) reflections on different components of the respective weight management interventions, (2) factors which influenced the participants’ commitment to the interventions and/or research, and (3) factors which influenced the participants’ motivation to engage with the interventions and/or research, see Figure 1.

**Theme 1: Components of the weight management intervention.** Participants reflected on how the different components of an intervention influenced their engagement. Participants commonly referred to goal setting, monitoring and peer support.

**Goal setting** was identified as a useful feature in supporting their progress. For example, participants noted the following:

‘[I am] Very engaged at the moment, driven by a goal to hit a target weight for the summer holidays’

‘With motivation for an end goal [I] can be very engaged and focused’.

Though the benefits of goal setting were limited. Participants reported that their motivations to engage reduced once the goals had been met. For example, participants reported that

‘[I] hit the goals, then stopped’

‘gained it all back’.

**Monitoring** was described as a means to track progress and was achieved using an array of methods. As described by participants, this included the following:

‘I monitor my step count and try and slowly improve on that’

‘Progression has been monitored not by weight but by photographs to get an impression on the loss of body fat’

‘watching my calorie intake and walking a bit more to offset some calories’

‘I judge my weight loss/gain by how I look and feel’

In addition to the reporting of how participants monitored their progress, some also gave an indication of time, highlighting that ‘Seeing results week on week keeps me motivated’.

**Peer support** was recognized as a feature which helped to promote engagement. Peer support was accepted in a variety of formats, including group support, individual, online or face-to-face.

‘Having a Facebook group and watching others progress helped motivate me’

‘talk to others is what keeps me on track with the plan’

Likewise, the type of support received was also reported to occur in different forms, this could be to have others motivating the participant, or the participant being motivated by other people’s success.

‘Seeing others being successful drives me to be successful’

‘my engagement this time is helped by the fact that my fiancé is also following the plan, its easier doing it together rather than on my own!’

‘worked out 2 or 3 times a week with friends’

**Theme 2: Commitment and Capability.** Participants described several factors which provided the opportunity to engage with the intervention and research. These factors included being held accountable, personalization and impact on lifestyle.

**Capability** was identified as influencing engagement. Participants reported different forms in which capability could present, highlighting health, knowledge, and time as specific factors, which were often identified as barriers, hindering engagement.

‘me not having the knowledge of cooking so I would be tempted to eat out.’

‘being too busy too follow [a plan] strictly’

‘Factors were the time available to go to the gym (work commitments getting in the way)’

**Commitment to research.** Similarly to the capability-related factors, these were presented as hindering engagement with research, whereby participants often reported lacking the opportunity to engage regularly and consistently, around their other commitments.

‘working a variety of different shifts on a weekly basis and having other life plans’

‘Too busy to follow strictly’

‘frequency of engagement, amount of time required to commit to the research’

In addition to the time commitment, participants also held their own perceptions of research which seemingly influenced their decision to participate in research. These included concerns around participation, and what they would gain from the research.

‘wanting to keep it private’, timing and beliefs about the research. For example, participants reported:

‘The fact that this is for academic research, I need it to feel useful. I am quite content in general and have a good relationship with food’.

Despite this, participants did consider the benefits of research participation. It was suggested that research could play a role similar to that of peer support in weight management interventions, whereby the research would hold the participants accountable to their engagement and progress, with the belief that the participants may be more likely to achieve their goals. Participants expressed this similarly throughout their responses, i.e.,

‘I would probably stick to the plan more’

‘having someone monitoring my progress from a research point of view would probably increase my engagement knowing they were seeing progress or not. it would give me another reason to stick to any plan rather than go it totally alone’.

**Theme 3: Motivation.** Participants acknowledged that there were both internal and external contributing factors which helped encourage them to remain engaged with the intervention and research. This primarily concerned the emotional experience and intrinsic motivators.

Regarding the **emotional experience,** participants appreciated weight management approaches which elicited a positive emotion such as enjoyment, which were more likely to maintain their engagement. This could be in response to the individual components of the approach as well as the approach itself. For example, participants reported the following:

‘I liked that I didn’t have to give up foods I loved’.

‘I started to enjoy working out and eating 200 g of veggies a day, I struggled a lot but slowly I started to like the different types of veggies’

On the contrary, negative emotions were noted to hinder engagement.

‘My engagement changes with how I’m feeling. If I feel good and positive I’m more likely to eat healthily and take exercise. The opposite is Also true.’

‘I stopped going due to feeling judged, I’m currently not following a weight loss plan’

‘I found it rather poor and repetitive’

‘[I tend] to eat more if tired, feeling a bit down etc.’

**Motivation for research.** Emotional experiences were similarly linked to engagement in research, as negative emotions once more hindered engagement. Participants reflected on their previous experiences, highlighting internally driven factors, including:

‘Putting to much pressure on myself and feeling a failure’

‘[losing] interest’

**Intrinsic motivators** reflected the participants’ personal aspirations for meeting their weight goals. This was personal to the participant, including, but not limited to, good health and wearing different types of clothing. For example:

‘At first I was fully engaged as it was for health reason’s. My anxiety was at a all time high because of my weight and I decided to do something about it’.

‘Tight clothing and nice clothes in my wardrobe are the driver for me’

However, these personal factors were described as a malleable constructs, subject to change depending on how well the participants could adhere to the intervention and whether the interventions met their expectations. To demonstrate this, one participant reflected on their progress:

‘[I] Hit goals but probably took longer than planned, lapsed from plan occasionally’.

Other participants reported similar experiences:

‘Fairly engaged but easily lose motivation to continue’

‘Working shifts e.g., nights, has a big impact on effort and motivation’

‘[I] had a weekend off and it all went to pot!’

**Motivation for research.** Participants considered how research would help them to stay motivated with the weight management intervention.

‘I think it would motivate me more’

‘I have already accomplished what I intended’

‘Taking part in research would definitely have a positive effect on my level of engagement’

However, this was caveated by the requirement to have direct benefits to the participant. Participants reflected on personal interests and contributions to their weight management experience.

‘The results I see in myself over time & the support’

‘having someone to motivate me’

‘If there was something looking at genuine psychological factors, looking at being over weight and obese in the same way that other eating disorders are looked at. I think that for a lot of people, it is deeply psychological. I wouldn’t be interested in any kind of faddy diet again’

## 4. Discussion

Due to consistent reports of attrition from weight management interventions and research, this study was conducted to explore factors which influenced engagement across a diverse range of approaches. As a result of our qualitative analyses, we identified three themes. Participants described components of weight-loss interventions as well as commitment-related and motivational factors which influenced their engagement with both the intervention itself and research associated with an intervention. Participants also discussed how research might affect engagement with a weight-loss intervention for both better and worse.

Consistent with McVay et al. (2018), commitment-related factors such as day-to-day scheduling and events that conflicted with planned activities, whether these were self-set if going it alone or part of an organized intervention, were a key barrier to engagement. Indeed, time is a factor consistently mentioned across behaviour-change models; for example, in the influential COM-B (Capability, Opportunity, Motivation and Behaviour) model, time is considered under the component ‘opportunity’ and in the model of user engagement for online behaviour change interventions (Short et al., 2015; West & Michie, 2020), time is considered as part of the ‘environment’ that affects engagement with an intervention. Notably, though, some of our participants took a converse view and felt that the timing of events, in particular, could be used as an external target for weight-loss goals. This observation highlights the importance of incorporating sporadic events into plans, to avoid conflict.

Our participants also identified a number of motivational factors that affected their engagement; this list included enjoyment of aspects of an intervention facilitating engagement and negative experiences (including feeling judged) making disengagement more likely. Indeed, ‘affect’ is a factor mentioned within the model of user engagement for online behaviour change interventions (Short et al., 2015). This finding supports and adds to the view that weight stigmatization in the context of weight-loss intervention is counter-productive (Wott & Carels, 2010), especially when internalized (Pearl et al., 2020).

Digital interventions are an important approach to increasing engagement with weight-loss interventions. A recent study used group videoconferencing to actively overcome personal and situational barriers to engagement with conventional weight management approaches (Cliffe et al., 2021). They found that this approach reduced the barriers mentioned in the current study centred around time and experience of judgement/stigma but preserved the valued intervention component of peer support, though the authors note that provision for technical difficulties had to be accounted for.

One of the key contributions of the current study is the consideration of engagement with research on weight-loss interventions. This showed that for those who were willing to participate in research, it could be a unique opportunity to increase intervention engagement. However, there was also an acknowledgement that this willingness to engage was contingent on the continuing benefit to the individual. Future studies may consider how such research can maximize benefit to the participant in order to preserve engagement.

The questionnaire-based approach was both a strength and limitation of the current study. On the one hand, this approach, alongside broad eligibility criteria, allowed for a large sample with a breadth of approaches to weight-loss intervention. However, there was no opportunity to ask follow-up questions to encourage participants to elaborate, and allowing participants to self-define the time period of “recent” weight loss may subject the findings to recall bias. Therefore, future research should consider implementing the engagement recommendations highlighted in this research to foster engagement in interviews to explore these themes further. Using an interview approach allows for more nuanced information to be gained, as the research can seek to clarify and check the accuracy of the subject’s responses (Braun & Clarke, 2006). Future research in this area may consider a more purposive approach to sampling, including, for example, the consideration that there has been a rapid increase in the use of obesity medications, namely GLP-1 agonists, though it has been noted that adherence can fluctuate, warranting further investigation to clarify the reason(s) behind this (Sharma et al., 2025).

It is of note that the majority of our participants identified as female, which limits generalizability to other populations. This is likely to be important in the current context given findings from Elliott et al. (2020) suggesting that gender-specific barriers may exist, including the perception of weight-loss services being a feminized space.

The ongoing challenge with this kind of work is that it is difficult to conduct research with those individuals who are very disengaged. Indeed, here, only 3% of our participants described themselves as ‘disengaged’ and 1% of our participants described themselves as ‘strongly disengaged’. Future research must continue to find inventive ways to identify barriers and facilitators to engagement with weight-loss interventions for those individuals who are least likely to engage in the first place. One recommendation to achieve this is through quantitative research to compare how level of engagement impacts engagement across different types of interventions.

Despite the ongoing rise in the prevalence of obesity, engagement in weight management interventions is variable. Drawing from existing interventions, it has been found that certain factors promote engagement, including personalized approaches, access to social support, the use of self-monitoring and receiving feedback. However, to further develop and demonstrate the effectiveness of weight management interventions, it is necessary for participants to engage with the research, yet engagement here is also variable.

## 5. Conclusions

Weight management interventions and research are subject to ongoing challenges with participant engagement and retention. Implementing a qualitative approach, this study aligns with established models of intervention and engagement, identifying several factors (i.e., goal setting, peer support and aligning to intrinsic motivation) to embed in both intervention and research design to enhance participant retention. In addition to addressing these factors, due to the findings, this study suggests that an effective approach may involve a greater integration of research with weight management interventions to promote engagement and reduce the burden on the participant. Therefore, it is recommended that approaches be designed in a more informed and integrated manner, as it is anticipated that this will facilitate participant engagement.

## Figures and Tables

**Table 1:** Participant demographic information.

Demographic	*M* (*SD*)	Total Participants
Gender		
Female		85 (87%)
Male		12 (12%)
Did not disclose		1 (1%)
Age	39.9 (13.9)	
BMI	29.32 (6.65)	

**Table 2:** Question text for open-ended questions and closed-ended questions quantifying engagement.

No.	Question Text	Response Format
1	How engaged were you with your weight loss plan and what factors/circumstances do you think affected your engagement?	Free-text response.
2	If you could rate your general engagement with your weight loss plan on this scale, how would you rate yourself? From	7-point scale: Strongly engaged, Engaged, Somewhat engaged, Neither engaged nor disengaged, Somewhat disengaged, Disengaged, Strongly disengaged.
3	Have you ever been a part of any kind of weight loss research?	3 options: Yes, No, Can’t remember.
4	If you haven’t, would you be willing to take part in research?	3 options: Yes, No, Have previously completed weight-loss research.
5	What do you think would affect your engagement (i.e., saying yes to taking part and sticking with the study over time) with research on your weight loss plan?	Free-text response.

**Table 3:** Frequency of responses per approach to weight management.

Primary Approach	Frequency	Percentage
Mainstream commercial weight-management programme	35	36%
Dietician-led	1	1%
Exercise programme (e.g., programme provided by gym)	8	8%
Going it alone (e.g., trying to eat less ‘junk’)	41	42%
Meal replacement	2	2%
Non-commercial diet plan (e.g., a plan without a subscription)	4	4%
Online support group	1	1%
Weight-loss app (excluding those provided as part of a broader commercial plan)	3	3%
Missing	3	3%
Total	98	100%

**Table 4:** Frequency of responses regarding participant engagement.

Level of Engagement	Number of Participants	Percentage
Strongly disengaged	1	1%
Disengaged	3	3%
Somewhat disengaged	10	10%
Neither engaged nor disengaged	13	13%
Somewhat engaged	26	27%
Engaged	33	34%
Strongly engaged	12	12%

## Data Availability

Data are not publicly available due to participant privacy and ethical restrictions. Anonymized data that support the findings of this study are available, on reasonable request, from the corresponding author.
